# Individual stability of single-channel EEG measures over one year in healthy adults

**DOI:** 10.1038/s41598-025-13614-y

**Published:** 2025-08-04

**Authors:** Tuuli Uudeberg, Laura Päeske, Hiie Hinrikus, Jaanus Lass, Toomas Põld, Maie Bachmann

**Affiliations:** 1 Biosignal Processing Laboratory, Department of Health Technologies, School of Information Technologies, Tallinn University of Technology, Ehitajate tee 5, 19086 Tallinn, Estonia; 2 Meliva AS, Rävala pst 5, 10143 Tallinn, Estonia

**Keywords:** Neuroscience, Biomarkers

## Abstract

The clinical applicability of electroencephalography (EEG) relies on the reliability and temporal stability of its measures. While the reliability of linear EEG measures is well established, the long-term stability of both linear and nonlinear measures at the individual level, as well as interindividual variability, remains underexplored. This study evaluated the one-year stability of EEG absolute band powers (theta, alpha, beta, and gamma) and nonlinear measures (Higuchi’s fractal dimension, Lempel–Ziv complexity, detrended fluctuation analysis, and in-phase Matrix Profile) across 12 monthly EEG recordings in nine healthy males aged 26–49. Intraclass correlation coefficients (ICCs) indicated excellent reliability across all measures, although beta power showed slightly reduced ICCs in temporal regions and gamma power demonstrated lower reliability in peripheral sites. At the individual level, nonlinear measures showed greater temporal stability than EEG band powers. Although a few individuals, particularly in band power measures, exhibited annual fluctuations comparable to or exceeding interindividual variability, most participants demonstrated consistent EEG profiles over time. These findings support the use of nonlinear EEG measures in longitudinal research and indicate their potential for developing personalized EEG-based neural biomarkers. They also highlight the importance of estimating expected individual variability when designing individualized monitoring approaches, as high reliability at the group level does not preclude substantial within-subject variability in some cases.

**Supplementary Information:**

The online version contains supplementary material available at 10.1038/s41598-025-13614-y.

## Introduction

Mental health disorders affect nearly one billion individuals worldwide, with anxiety and unipolar depression being among the most prevalent conditions, impacting approximately 580 million people^1^. Mental health conditions constitute a leading cause of disability, and the COVID-19 pandemic further exacerbated their global burden, leading to a 25% increase in anxiety and depression cases due to social isolation, financial distress, and health-related concerns^1^. Despite their prevalence, mental disorders remain significantly undertreated, with 75% of individuals in low- and middle-income countries receiving no treatment due to resource limitations, stigma, and systemic barriers^1,2^. Furthermore, the diagnosis and treatment of mental health disorders remain largely subjective, relying on clinical interviews and self-report questionnaires. These methods introduce variability due to the respondents’ willingness and ability to comprehend and answer questions, clinicians’ expertise, and sociocultural factors, resulting in frequent misdiagnosis and inadequate treatment access^2^.

Changes in mental health are reflected in alterations in brain activity. Electroencephalography (EEG) is an effective complementary method to traditional clinical assessments for evaluating mental health, offering an objective and cost-effective tool for capturing electrical activity generated by cortical neurons near the scalp. EEG provides quantifiable measures that can aid in early diagnosis, track disease progression, and evaluate treatment efficacy^3^. EEG’s affordability, high temporal resolution, and non-invasive nature make it a valuable tool for investigating brain dynamics in both clinical and healthy populations. Over the decades, EEG has been widely utilized, leading to the development and adoption of various methods to compute different EEG measures for studying cognitive functions and neurological disorders^4–12^.

### EEG linear and nonlinear measures

Traditional EEG analysis relies on spectral band power measures, which provide essential insights into brain dynamics by quantifying neural oscillations across different frequency bands. EEG frequency bands are linked to distinct cognitive and physiological processes, with delta (0.5–4 Hz) associated with deep sleep, theta (4–8 Hz) with memory and drowsiness, alpha (8–13 Hz) with relaxation and attentional control, beta (13–30 Hz) with active thinking and motor planning, and gamma (> 30 Hz) with higher-order cognitive functions such as perception and consciousness^3^. While band power and other linear measures have been extensively studied, they do not fully account for the dynamic and complex nature of neural activity^3^. As the brain operates as a nonlinear system, nonlinear EEG measures have been developed or adapted from other domains to capture its self-organizing dynamics better. These methods provide additional information to linear measures by quantifying irregularity, complexity, and long-range temporal dependencies in neural signals. Probably the most used complexity measures are fractal dimensions. Higuchi’s fractal dimension (HFD) estimates the self-similarity of EEG signals, reflecting neural complexity, and has been applied to many different areas of neurological and mental health research^6,8,12–16^. Detrended fluctuation analysis (DFA) measures long-range temporal correlations (LRTC)^17,18^ and has also been successfully applied in EEG studies^4,7,10^ as well as Lempel–Ziv complexity (LZC) that measures the number of new patterns in a time series^19,20^. A more recent method, the in-phase Matrix Profile (pMP), has been introduced to identify repeating patterns in EEG signals^12^. The in-phase Matrix Profile adapts the fast Matrix Profile similarity-search algorithm^21^ to EEG by comparing fixed-length, phase-aligned subsegments by calculating Euclidean distances, yielding a parameter-free index of segment-to-segment self-similarity in the time domain. Its first EEG application outperformed HFD in distinguishing patients with major depressive disorder from healthy controls, underscoring the method’s diagnostic potential^12^. Bachmann et al.^8^ demonstrated that combining linear and nonlinear EEG measures improves classification accuracy when distinguishing depressed individuals from healthy controls, reinforcing the potential utility of these measures in clinical applications. Although some nonlinear EEG methods have been used for decades, their potential still remains underexplored compared to traditional spectral approaches. Given that nonlinear methods align more closely with the brain’s intrinsic dynamics, richer information about neural function and dysfunction is expected.

For the present single-channel resting-state design, we restricted the nonlinear feature set to four time-domain measures (HFD, DFA, LZC, and pMP) because together they span scale-free complexity, long-range temporal correlations, algorithmic irregularity, and segment-to-segment in-phase self-similarity while requiring little or no parameter tuning. Entropy-based alternatives (e.g., sample or permutation entropy) were not included, as their reliability depends strongly on embedding and tolerance parameters and on longer stationary epochs, which can hamper longitudinal comparability^22–24^.

### Reliability of EEG measures

For EEG measures to be effectively utilized in clinical and research applications, they must demonstrate high reliability and temporal stability. Establishing temporal stability in EEG measures is essential to distinguish genuine brain-state-related neural changes from intrinsic EEG variability. Stable EEG measures enhance the validity and interpretability of findings, thereby improving clinical decision-making and advancing scientific understanding of brain function and disorders.

The reliability of linear EEG measures, particularly power in standard frequency bands, has been well studied, with early studies confirming the reliability and stability of power across different frequency bands^25–27^. More recent investigations have expanded on these findings by examining the reliability of additional linear measures^28–31^. However, considerably less research has focused on the reliability and stability of nonlinear EEG measures. Only a few studies have included them in their analyses^15,16,32,33^. The available evidence suggests that nonlinear measures exhibit either lower reliability than traditional EEG band power measures^32,33^ or a level of reliability comparable to linear measures^15^, indicating that these measures may capture aspects of EEG dynamics not reflected in the power of traditional frequency bands.

Gudmundsson et al.^33^ investigated the stability of quantitative EEG measures in 15 healthy elderly individuals over two months (19 EEG recordings per participant). Their findings indicated that band power measures demonstrated the highest reliability, with mean ICCs of 0.77 for absolute power and 0.80 for relative power across eight channels and all frequency bands. Complexity-based measures such as LZC exhibited lower reliability (ICC = 0.70), while coherence measures were the least stable, with their reliability strongly dependent on channel location.

Põld et al.^15^ conducted a three-year test–retest study on 17 healthy participants, reporting that relative power measures exhibited reliability comparable to nonlinear measures such as HFD and DFA. The highest reliability was observed for relative alpha power (mean ICC = 0.87 across 18 channels). Although ICCs for EEG frequency bands and nonlinear measures were comparable, the nonlinear measures demonstrated greater temporal stability at the group level, as reflected by smaller relative differences between the two recordings. Lord & Allen^16^ studied 306 subjects, including controls and individuals with a history or current episode of depression, and found high internal consistency for HFD and sample entropy within single sessions, as well as high reliability across multiple days (ICCs for HFD ranging between 0.64 and 0.86 across different channels in eight recording sessions conducted over four days within two weeks).

Despite these contributions, existing studies provide limited understanding of EEG temporal stability at the individual level. Many studies employ test–retest designs with only a few EEG recordings per participant^15,26–28,30–32^, while others cover short observation periods of up to two months^16,33^. While these studies offer valuable insights into EEG stability, they do not consider the characteristics of individual participants.

### Person-specific EEG patterns

Numerous studies have successfully distinguished between a control group and a group with mental disorders using both linear and nonlinear EEG measures^8–12^. However, although these group-level results are promising, a measure that separates diagnostic groups may still reveal little about within-person EEG variability and thus may not capture clinically meaningful deviations in an individual over time.

Brain activity patterns are expected to exhibit strong individual specificity^34^, and EEG signals have been suggested to function as a unique neural fingerprint^35,36^. However, for EEG measures to be effective in detecting neural changes within individuals, it is essential first to establish their normal variability in a healthy state, as this variability is expected to differ from individual to individual. Without a clear understanding of this baseline variability, it remains difficult to determine whether a new measurement reflects normal fluctuations or a deviation indicative of altered brain function. Detecting such deviations assumes that EEG measures remain relatively stable within an individual under normal conditions. At the same time, excessive fluctuations may either lead to misinterpreting normal variability as pathological or cause true pathological changes to go unnoticed, thereby undermining the applicability of an EEG measure.

Although interest in individualized EEG analysis is increasing, longitudinal studies examining EEG stability at the individual level over extended periods remain limited. Previously, we conducted a single-participant case study evaluating EEG-based individual measures over 15 sessions spanning 14 months^37^. While this study provided valuable insights into the long-term stability of linear and nonlinear measures, inter-individual differences cannot be assessed based on a single subject. More extensive studies are needed to establish individual variability in the healthy state by determining the extent to which EEG measures remain stable within individuals over months or years.

The lack of longitudinal research at the individual level is a significant barrier to the clinical application of EEG. While EEG measures may exhibit high test–retest reliability and temporal stability, their long-term stability at the individual level remains largely unexamined. A dependable clinical measure should achieve an optimal balance between long-term stability, ensuring consistency across repeated measurements under similar conditions, and sensitivity to meaningful physiological changes over time. Understanding these dynamics of EEG variability is critical for both clinical and research applications, ensuring that EEG-based measures are applicable, interpretable, and reliable for individual-level diagnostics and monitoring.

### Study objectives

The aim of this study is to examine the temporal stability of single-channel EEG measures at the individual level over one year, based on repeated monthly recordings. While previous research has primarily addressed short-term test–retest reliability or group-level comparisons, this study focuses on individual consistency and variation over time in healthy adults. We assess both linear EEG measures (absolute power in theta, alpha, beta, and gamma frequency bands) and nonlinear measures (HFD, LZC, DFA, and pMP), evaluating their person-specific variability. Based on this framework, we formulate two hypotheses: (1) Although EEG measures differ between individuals, they remain temporally stable within the same person over one year. (2) Nonlinear EEG measures exhibit greater temporal stability at the individual level compared to absolute band powers.

By characterizing stable, person-specific EEG patterns and describing the typical range of variation observed for each individual, this study aims to support the development of individualized EEG biomarkers and contribute to future personalized monitoring approaches in mental health research.

## Methods

### Subjects

Nine healthy male subjects participated in the study. We restricted the sample to males to avoid menstrual-cycle–related variability, as resting-state neural oscillations have been shown to fluctuate across cycle phases in EEG^38^ and magnetoencephalography^39^. At the time of the first recording, participants had a mean age of 37.2 ± 8.1 years, with an age range of 26 to 49 years. All participants self-reported as right-handed, nonsmokers, and free of any history of concussions involving loss of consciousness, narcotic or psychotropic substance use, alcohol abuse, or mental or psychiatric disorders.

To ensure consistency, participants were instructed to maintain their usual daily routines and refrain from consuming alcohol or caffeinated beverages for 24 h before each recording. The study was conducted following the Declaration of Helsinki and received formal approval from the Tallinn Medical Research Ethics Committee and the Estonian Institute for Health Development’s Human Research Ethics Committee. All participants signed written informed consent before the study.

### Collection of EEG data

For each participant, EEG recordings were scheduled every four weeks (with flexibility for five to six weeks in exceptional cases, such as illness or travel), resulting in a total of 12 recordings over the course of one year. Recordings were conducted on a consistent day of the week and at the same time of day, ensuring homogeneity. To minimize dietary influences on EEG activity, all recordings took place in the morning, with participants instructed to abstain from eating or drinking (except water) beforehand^40^.

**Fig. 1:**
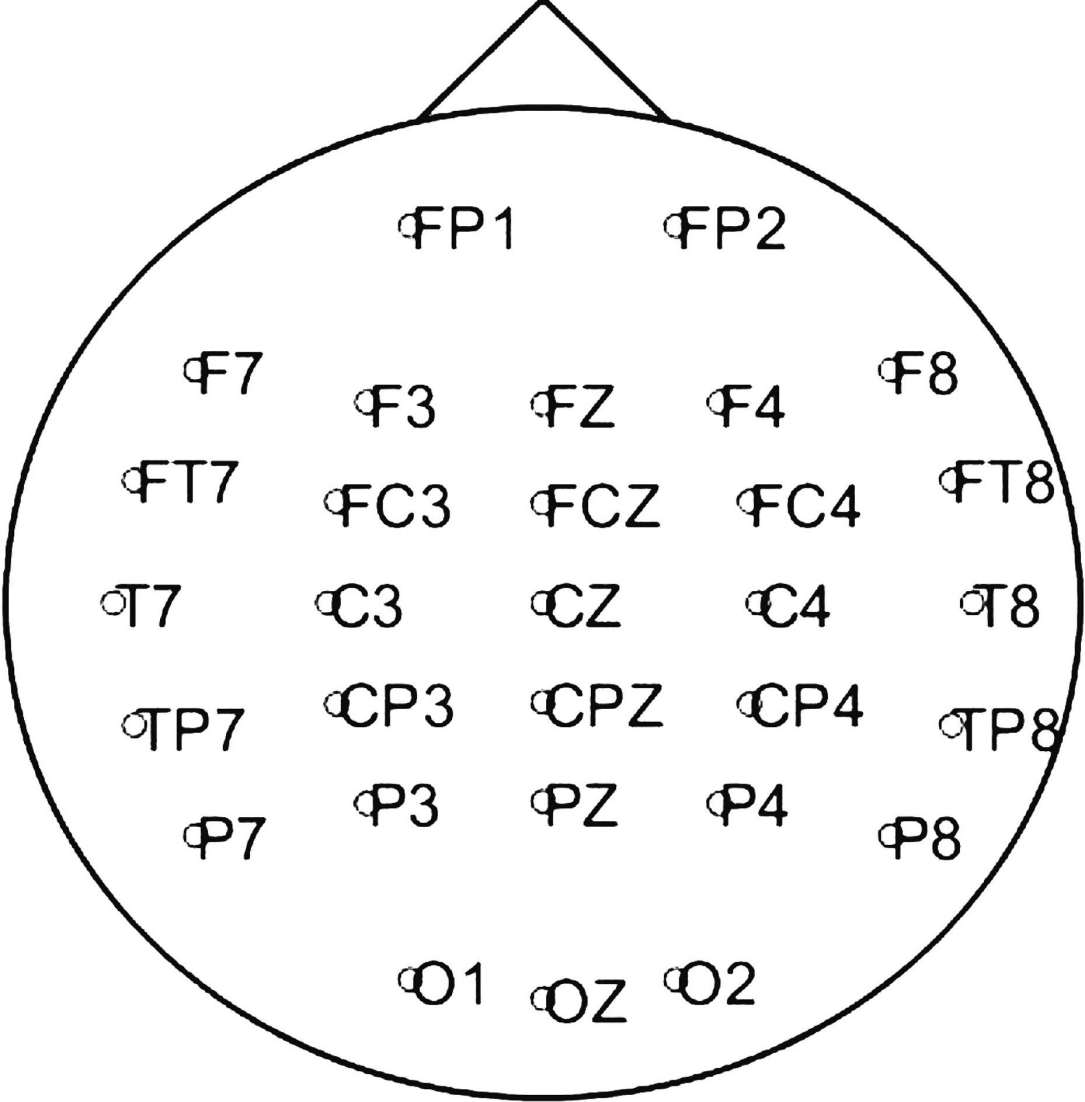
Locations of the 30 EEG electrodes corresponding to the channels used in this study, positioned according to the extended international 10/20 system.

EEG data were collected using the Neuroscan Synamps2 acquisition system and a 32-channel (30 EEG + 2 EOG) Quick-Cap (Compumedics, NC, USA). Electrodes were positioned according to the extended international 10/20 system, with linked mastoids as reference. The placement of the 30 EEG electrodes is shown in Fig. 1.

During recordings, participants were lying in a relaxed supine position in a dimly lit laboratory room. EEG was recorded for 10 min with eyes closed and 5 min with eyes open across 30 EEG channels. Electrode impedance was maintained below 10 kΩ. EEG data were recorded at a sampling rate of 1 kHz, within a frequency range of 0.3–200 Hz.

### EEG data preprocessing

All calculations were performed using MATLAB software (The MathWorks, Inc.). Initially, the eyes-closed EEG recordings were divided into 20.48-second segments, and segments with apparent artifacts were identified through visual inspection. Next, the full eyes closed EEG data were re-referenced using the Reference Electrode Standardization Technique (REST), which is a reliable method for low-density EEG montages and facilitates comparability across laboratories^41–43^. To remove baseline fluctuations and high-frequency noise, Parks–McClellan forward-backward filters were applied, yielding a frequency bandwidth of 2–47 Hz for further analysis. Absolute power in each frequency band was computed using the original sampling rate of 1 kHz, while EEG data were downsampled to 200 Hz for nonlinear measure calculations. After filtering (and downsampling), the EEG signals were divided into 20.48-second non-overlapping segments again, with segment lengths defined as 20,480 samples (for absolute power calculations) and 4096 samples (for nonlinear measures). The first 12 clean segments were selected for the computation of the following EEG measures, resulting in 12 values for each measure per subject and per recording session.

### Calculation of EEG measures

#### Absolute power of EEG frequency bands

The absolute power *P* for each frequency band was calculated directly from a filtered EEG signal using a time-domain approach. Each frequency band was first extracted using a Parks–McClellan forward-backward bandpass filter. In this study, the absolute powers of the traditional theta (4–8 Hz), alpha (8–13 Hz), beta (13–30 Hz), and gamma (30–47 Hz) frequency bands were calculated for each subject according to

1 $$\:P\:=\:\frac{1}{n}\sum\:_{i=1}^{n}{x}^{2}\left(i\right),$$

where *x(i)* was the filtered EEG signal segment with the length *n* (20,480 samples) at sample *i*.

#### Higuchi’s fractal dimension

HFD is used to quantify the complexity of EEG signals, providing a measure of scale invariance or self-similarity across multiple temporal scales. The method, originally proposed by Higuchi^14^, estimates the fractal dimension within the interval [1,2] of a time series by analyzing its length at different scales. A higher HFD value indicates greater signal complexity, while a lower value reflects more regular and predictable neural activity. The HFD was calculated with the parameter *k*_*max*_ = 8 according to the algorithm presented in^14^. For the calculation of HFD for an EEG signal segment with the length *n* (4096 samples), a time series $\:{X}_{k}^{m}$ is formed for each scale factor *k* as in

2 $$\:{X}_{k}^{m}=\:\left\{x\left(m\right),\:x\left(m+k\right),\:x\left(m+2k\right),\:\ldots\:\right\},\:\:\:\:m=1,\:2,\:\ldots\:,\:k,$$

where *k* represents the step size and *m* is the starting index of each subseries. The length of each subseries *L*_*k*_*(m)* is calculated as in

3 $$\:{L}_{k}\left(m\right)=\:\frac{1}{k}\sum\:_{i=1}^{\left\lfloor\frac{n-m}{k}\right\rfloor}\left|x\left(m+ik\right)-x(m+\left(i-1\right)k\right|\cdot\:\frac{n-1}{k\,\left\lfloor\frac{n-m}{k}\right\rfloor},$$

The${\,\left\lfloor\frac{n-m}{k}\right\rfloor}$ term $\:\frac{n-1}{k\,\left\lfloor\frac{n-m}{k}\right\rfloor}$ normalizes the subseries length and ensures that the length $\:{L}_{k}\left(m\right)$ is expressed by the average number of points in the subseries and therefore comparable across all scale factors *k* (see^44^ for a step-by-step illustration). $\:{L}_{k}\left(m\right)$ The mean length for each *k* is obtained by averaging across all subseries as in

4 $$\:L\left(k\right)=\:\frac{1}{k}\sum\:_{m=1}^{k}{L}_{k}\left(m\right).$$

The fractal dimension is estimated by fitting a linear regression line to the logarithmic plot of $\:L\left(k\right)$ versus 1/*k*, where the slope of this resulting log–log line corresponds to Higuchi’s fractal dimension.

#### Lempel–Ziv complexity

LZC is a measure of sequence complexity, quantifying the rate at which new patterns emerge as a sequence progresses^19^. It is used to assess signal randomness and complexity, with higher LZC values indicating more irregular and complex signals, while lower values suggest more repetitive or structured patterns. To calculate LZC, first, EEG signal segment *x(i)* of length 4096 samples is binarized into $\:{B}_{i}$ using threshold *T*, which in this study was the median of the EEG signal segment to minimize the impact of outliers. Samples below the median get a new value of zero, others one as in

5 $$\:{B}_{i}=\:\left\{\begin{array}{c}1,\:\:if\:{S}_{i}\:\ge\:T\\\:0,\:\:if\:{S}_{i}\:<T\end{array}\right. .$$

Second, the binary sequence is scanned from left to right to find new patterns. A new pattern is detected whenever a substring is encountered that has not appeared previously in the sequence during left-to-right parsing. The complexity counter $\:C\left(n\right)$ increases each time a new pattern is encountered. Finally, LZC is normalized to avoid variations due to segment length as in

6 $$\:{LZC}_{norm}=\:\frac{C\left(n\right)}{{C}_{max}\left(n\right)},$$

where $\:{C}_{max}\left(n\right)$ is the theoretical maximum complexity for a completely random sequence of length *n*, approximated as $\:n/{\text{l}\text{o}\text{g}}_{2}\left(n\right)$. This normalization ensures that LZC values range between 0 (completely regular signal) and 1 (maximally complex, random signal).

#### Detrended fluctuation analysis

DFA was calculated according to the method described by Peng et al.^17,18^. First, the cumulative sum of the mean-centered EEG signal segment *x(i)*, with the length of *N* (4096 samples) was calculated to generate an integrated time series as in

7 $$\:y\left(k\right)=\:\sum\:_{i=1}^{k}\left[x\right(i)\:- \bar{x} ],$$

where *k* gets a value from 1 to *N* and $\:\bar{x}$ is the arithmetic mean of the signal segment *x(i)*. Second, the integrated signal *y(k)* is divided into *n* equal nonoverlapping windows of a length ranging from 4 to 200 samples. In each window *n*, the local trend is estimated using a least-squares linear fit $\:{\widehat{y}}_{n}\left(k\right)$, which fits the data *y(k)*, and the local trend is subtracted from the data. Average fluctuations are given by

8 $$\:F\left(n\right)=\:\sqrt{\frac{1}{K}\sum\:_{k=1}^{K}{\left[y\left(k\right)-\:{\widehat{y}}_{n}\left(k\right)\right]}^{2}}.$$

Here, *K* is the number of nonoverlapping windows of length *n*. These average fluctuations are calculated for all window lengths. A log-log plot of *F(n)* versus *n*, reveals a linear scaling, characterized by the slope of the line, which represents the scaling exponent α. This exponent reflects the presence and strength of long-range temporal correlations in the signal: *α* = 0.5 indicates white noise (no correlation), while *α* > 0.5 suggests persistent correlations.

#### In-phase matrix profile

The pMP method captures the self-similarity of the EEG signal by considering only the in-phase subsegments, making it sensitive to the periodicity of alpha waves and other frequency fluctuations in the EEG signal. First, a one-second subsegment (200 samples) was extracted from a 4096-sample EEG segment, and its Euclidean distance to all other subsegments within the same segment was calculated, generating a distance profile (DP). This process was then repeated for the next subsegment, continuing in a sliding window manner until a DP was obtained for each subsegment.

From each DP, the smallest Euclidean distances corresponding to the most in-phase subsegments were extracted. In-phase subsegments are defined as those with minimal phase shift and the highest waveform similarity to the reference window, based on time-domain Euclidean distance. The median of these in-phase distance values was then calculated for each DP, forming a pMP vector (pMPvec). The median was used to ensure robustness against outliers in the signal.

Finally, the mean of the pMPvec is computed to obtain the overall pMP value for the EEG segment. This value reflects the degree of temporal regularity and self-similarity in the signal, where lower values indicate more consistent recurring patterns.​ The calculation process is explained in detail in^12^.

### Statistical analysis

Since we calculated 12 values for each measure for every EEG channel in each recording of each participant, we subsequently used the median of these 12 values.

With 12 monthly recordings for nine subjects, we utilized the intraclass correlation coefficient (ICC)^45,46^ to assess the reliability of repeated EEG measurements. ICC quantifies the proportion of total variance attributable to differences between subjects, providing a measure of the stability and consistency of EEG measures over time. When applied to datasets with multiple measurements per subject, ICC evaluates the degree of agreement among repeated observations within individuals relative to overall variability. A high ICC indicates that an EEG measure is relatively consistent within individuals across repeated sessions and shows greater variability between individuals than within individuals. We employed a two-way mixed-effects model (average measures, absolute agreement)^45,47^ for all 30 channels, ensuring that both systematic subject differences and measurement error were accounted for in assessing temporal stability.

ICC was calculated as in

9 $$\:ICC=\:\frac{{MS}_{R}-\:{MS}_{E}}{{MS}_{R}+\:\frac{{MS}_{C}-\:{MS}_{E}}{n}},$$

where *MS*_*R*_ is the mean square for subjects (i.e., between-subject variance), *MS*_*C*_ is the mean square for repeated measurements (i.e., between-measurement variance), *MS*_*E*_ is the mean square error, and *n* is the number of subjects.

We employed the Kruskal–Wallis test (α = 0.05) for data analysis^48^. The Kruskal–Wallis test is a nonparametric alternative to ANOVA to determine whether there are significant differences between three or more groups (in this case, subjects). Unlike ANOVA, the Kruskal–Wallis test does not assume a normal distribution of the data and is not sensitive to unequal variances. If a significant difference is detected between any of the subjects, a post-hoc test can be conducted to determine which subjects are different from each other. In this study, we employed the Dunn test (α = 0.05) to determine how many subject pairs were statistically different from each other^49^. As with 9 subjects, we had 9(9 − 1)/2 = 36 unique pairwise comparisons, we used the Šidák correction^50^ of the probability (*p*) values as in

10 $$\:{p}^{*}=1-(1-p{)}^{m},$$

where *m* is the number of comparisons and $\:{p}^{*}$ is the corrected *p*-value.

For each participant, we calculated the annual mean and standard deviation for each measure, as well as the maximum relative difference ($\:rDif$), which indicates the largest deviation from the annual mean as in

11 $$\:rDif=\:\left|\frac{{v}_{max}-\:\bar{v}}{ \bar{v} }\right|*100,$$

where $\:{v}_{max}$ is the most extreme monthly measurement across the year for a given subject, and $\bar{v}$ is that subject’s annual average.

## Results

### Intraclass correlation coefficients for EEG measures

**Fig. 2:**
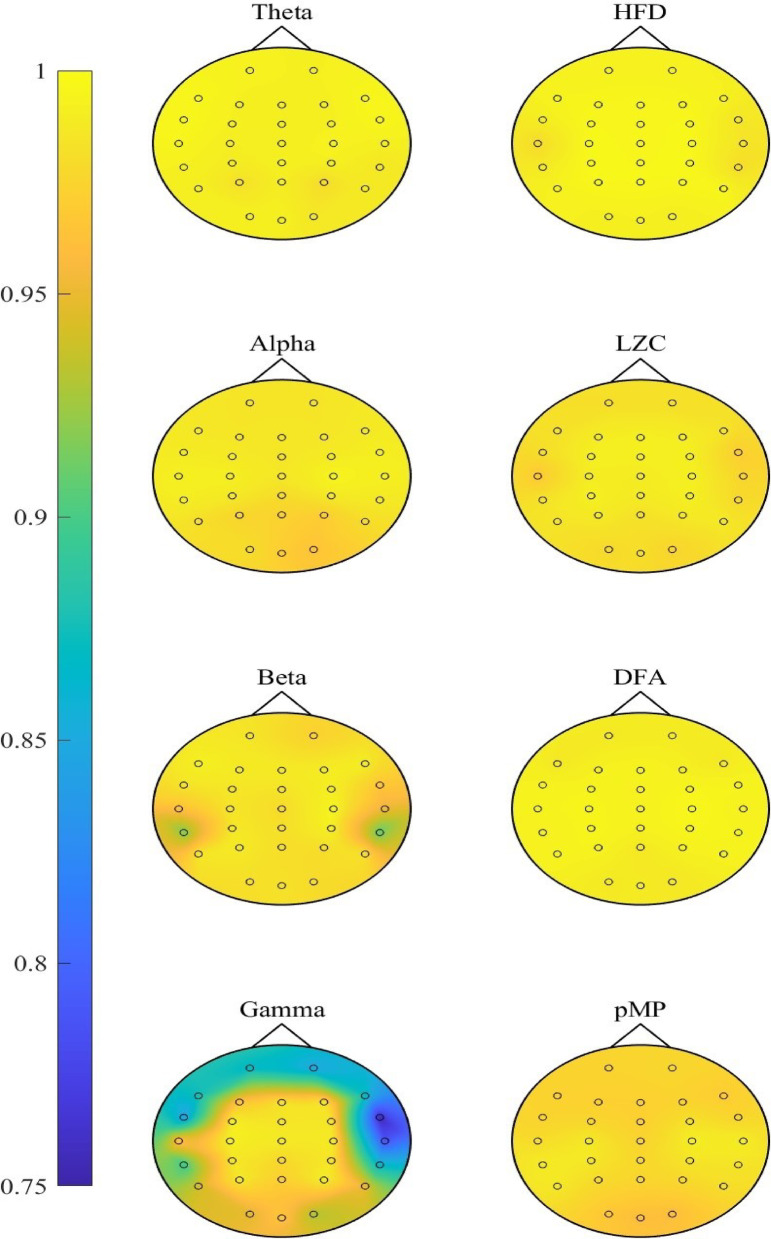
Intraclass correlation coefficients for EEG measures across all 30 EEG channels (*n* = 9), including theta, alpha, beta, and gamma absolute powers, as well as nonlinear measures: Higuchi’s fractal dimension (HFD), Lempel–Ziv complexity (LZC), detrended fluctuation analysis (DFA), and in-phase Matrix Profile (pMP).

Figure 2 presents the intraclass correlation coefficients (ICCs) for EEG band powers and nonlinear measures, while detailed ICCs for all EEG measures across all 30 channels are provided in Supplementary Table S1. Based on the classification proposed by Koo and Li^47^, we considered ICCs to indicate excellent reliability when the lower bound (LB) of the 95% confidence interval (CI) exceeded 0.9.

### Band power measures

The data in Fig. 2 demonstrate that lower-frequency EEG bands, theta and alpha, exhibited reliability classified as excellent over one year across all 30 EEG channels. The lowest ICCs were 0.979, 95% CI [0.952, 0.994] in P4 for theta power and 0.964, 95% CI [0.917, 0.990] in O2 for alpha power, indicating high reliability over time. Beta power demonstrated excellent ICCs across 27 channels, with slightly lower values in three temporal channels (TP7, T8, TP8). The lowest ICC was observed at TP8 (0.908, 95% CI [0.786, 0.975]), still indicative of good reliability. For gamma power, ICCs were excellent in 17 channels in the center of the head but lower in 13 peripheral channels, including the prefrontal area, with the lowest ICC at FT8 (0.756, 95% CI [0.424, 0.935]).

A slight reduction in ICCs observed in a few temporal channels in the beta band, and more notably lower ICCs across several peripheral channels in the gamma band, may be influenced by the presence of electromyographic (EMG) activity. EMG signals, resulting from muscle contractions, are commonly associated with movements such as swallowing, chewing, or speaking, but can also be present at a low level during resting state without overt motion^51^. Although relaxation can help minimize such activity, the spectral overlap between EMG and the beta and gamma frequency bands complicates the effective removal of these artifacts. EMG activity typically spans the 15–300 Hz range, with most power concentrated at the lower end^52,53^.

In this study, muscle artifacts related to conscious movement were excluded from the EEG recordings. However, some low-level muscle tension, which is difficult to detect through visual inspection, may have remained in channels positioned over the temporalis and frontalis muscles. Tonic muscle activity, referring to the continuous low-level contraction of muscles even in a relaxed state, can contribute to subtle EEG interference. Unlike phasic muscle activity, which is associated with voluntary movements, tonic muscle activity persists at a baseline level and can be influenced by factors such as posture, alertness, and individual muscle tone^51^. In EEG recordings, this may appear as low-amplitude, high-frequency activity, particularly in frontal and temporal regions where muscles like the frontalis and temporalis are located.

### Nonlinear measures

All nonlinear EEG measures (HFD, LZC, DFA, and pMP) exhibited excellent reliability (ICC 95% CI LB > 0.9) across all EEG channels (Fig. 2). The lowest ICC among these measures was observed for pMP, with a value of 0.960, 95% CI [0.908, 0.989] in the occipital channel Oz. LZC demonstrated a slightly higher ICC of 0.967, 95% CI [0.922, 0.991] in T7, while DFA and HFD showed the highest reliability with the lowest ICC of 0.986, 95% CI [0.967, 0.996] in O2 and 0.978, 95% CI [0.949, 0.994] in T7, respectively. Although pMP had slightly lower ICCs in the occipital region, they remained within the excellent reliability range. Given that pMP is influenced by alpha oscillations^12^ and alpha power is strongest in occipital areas, variability in alpha activity may have contributed to this observation.

While beta power showed slightly reduced ICCs in only a few temporal channels, gamma power exhibited more widespread reductions (ICC 95% CI LBs ≤ 0.9 in 13 channels), particularly in peripheral temporal, frontotemporal, and prefrontal areas. These reductions may, at least in part, reflect the potential influence of residual EMG activity. Nevertheless, given the overall excellent reliability across measures, any channel may be used for further analysis, while it may be advisable to avoid regions that are more prone to muscle-related influences.

### Individual variability of EEG measures

**Fig. 3:**
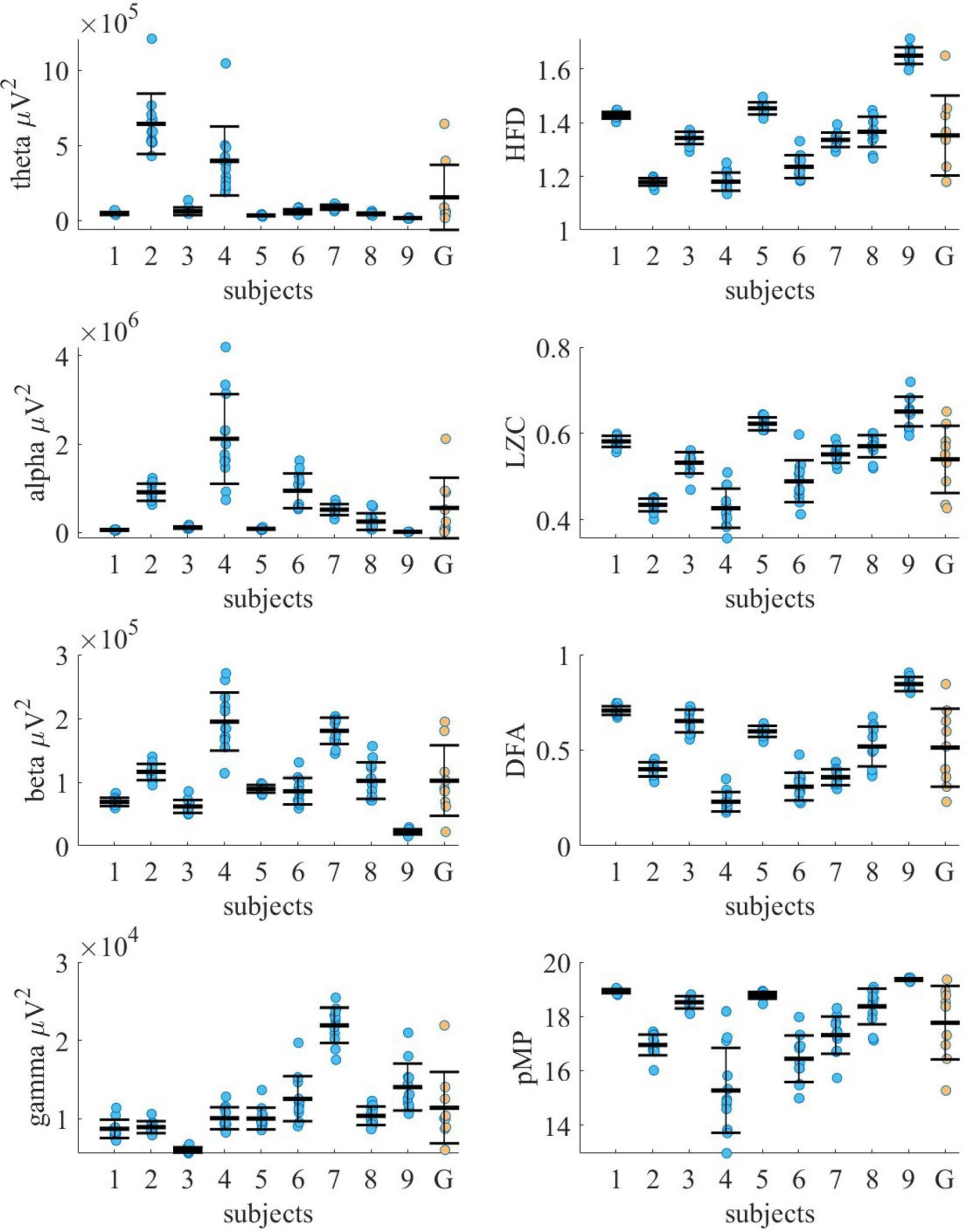
Interindividual and intraindividual variability in EEG measures across one year for each subject 1–9 and the group G (*n* = 9). Blue dots represent 12 individual monthly values; black dashes show subject-specific annual means. Error bars for subjects 1–9 represent within-subject standard deviations. For group G, yellow dots represent the annual mean of each subject, the black dash shows the group-level mean, and error bars indicate the standard deviation.

To investigate person-specific EEG dynamics over time, we examined the individual temporal variability of EEG measures across one year, as presented in Fig. 3; Tables 1 and 2. The figure displays the EEG measure values recorded throughout the year, along with the annual mean and standard deviation for the parietal channel P3. This channel was selected as an example due to its consistent reliability in resting-state EEG, low susceptibility to muscle artifacts, and its well-established role in reflecting stable, individual differences in neural activity, particularly within parietal regions involved in cognitive processing^54,55^.

**Table 1:** Annual mean values, standard deviations, and relative maximal differences from the annual mean (rDif, %) of theta, alpha, beta, and gamma absolute power calculated for nine subjects in channel P3 and for the group (*n* = 9).

	Theta			Alpha			Beta			Gamma		
	Mean^a^	SD^a^	rDif	Mean^a^	SD^a^	rDif	Mean^a^	SD^a^	rDif	Mean^a^	SD^a^	rDif
Subject 1	46.93	9.48	48	55.73	9.19	27	68.97	6.48	20	8.69	1.18	31
Subject 2	643.66	201.24	88	912.09	196.03	35	115.89	12.61	21	8.91	0.76	19
Subject 3	62.10	25.90	120	110.14	26.05	58	61.86	10.15	39	5.98	0.35	12
Subject 4	396.86	228.87	163	2119.96	1015.02	98	194.81	45.43	42	10.05	1.41	27
Subject 5	34.29	4.90	24	80.81	18.97	50	89.63	6.07	11	10.00	1.39	37
Subject 6	58.90	16.73	50	945.82	395.22	72	85.79	20.80	52	12.54	2.87	57
Subject 7	88.54	16.45	30	517.02	124.77	43	180.43	20.73	20	21.92	2.26	20
Subject 8	44.60	8.49	43	245.04	189.06	152	102.30	28.56	53	10.36	1.19	18
Subject 9	16.96	2.94	27	11.26	2.41	43	21.92	4.16	32	14.03	2.99	49
Group	154.76	217.10	316	555.32	688.71	282	102.40	55.33	90	11.39	4.56	92

^a^ Values must be multiplied by 10³ to obtain the correct magnitude in µV².

**Table 2:** Annual mean values, standard deviations, and relative maximal differences from the annual mean (rDif, %) of Higuchi’s fractal dimension (HFD), Lempel–Ziv complexity (LZC), detrended fluctuation analysis (DFA), and in-phase matrix profile (pMP) calculated for nine subjects in channel P3 and for the group (*n* = 9).

	HFD			LZC			DFA			pMP			
	Mean	SD	rDif	Mean	SD	rDif	Mean	SD	rDif	Mean		SD	rDif
Subject 1	1.43	0.01	2	0.58	0.01	4	0.71	0.02	5	18.93		0.08	1
Subject 2	1.18	0.01	3	0.43	0.01	8	0.40	0.04	16	16.95		0.38	6
Subject 3	1.34	0.02	4	0.53	0.02	12	0.65	0.06	15	18.52		0.22	2
Subject 4	1.18	0.03	6	0.43	0.05	19	0.23	0.05	52	15.28		1.56	19
Subject 5	1.45	0.02	3	0.62	0.02	4	0.60	0.03	9	18.77		0.13	2
Subject 6	1.24	0.04	8	0.49	0.05	22	0.31	0.07	54	16.44		0.86	9
Subject 7	1.34	0.03	4	0.55	0.02	7	0.36	0.04	22	17.31		0.69	9
Subject 8	1.37	0.06	7	0.57	0.03	9	0.52	0.10	30	18.37		0.65	7
Subject 9	1.65	0.03	4	0.65	0.03	11	0.85	0.04	7	19.36		0.05	0
Group	1.35	0.15	22	0.54	0.08	21	0.51	0.20	65	17.77		1.35	14

As illustrated in Fig. 3, EEG measure values for each subject fluctuate around a distinct annual mean, with variability ranges that are specific to the individual. These subject-specific patterns give rise to clearly separable clusters in the data, with the extent of variability differing across individuals. A Kruskal–Wallis test confirmed that at least one of the clusters was statistically different from the others for each measure (*p* ≤ 1.1 × 10^–15^).

Statistically significant differences were observed between 14 and 16 subject pairs out of 36 pairwise comparisons, depending on the measure, using Dunn’s test with Šidák *p*-value correction. There was no considerable difference in statistical significance between EEG band power and nonlinear measures. Specifically, theta power differed significantly in 15 pairs, alpha power in 16 pairs, and beta and gamma power in 14 pairs. Among nonlinear measures, significant differences were observed in 14 pairs for HFD and LZC, and in 15 pairs for DFA and pMP, out of 36 comparisons. These results indicate that, regardless of the type of measure, individual EEG profiles are characterized by distinct annual means and specific fluctuation ranges, supporting the idea of temporally stable neural individuality.

Compared to EEG frequency band powers, nonlinear EEG measures show relatively higher temporal stability on the individual scale (Tables 1 and 2). Among the band power measures, theta power shows the greatest individual fluctuation, with a single recording maximally differing from the annual mean by an average across all subjects of 66% (ranging from 24 to 163%, depending on the subject). This is followed by alpha power, which maximally fluctuates by an average of 64%, with individual variation ranging from 27 to 152%. Beta and gamma power exhibit lower variability, with average maximal deviations of 32% and 30%, respectively. Individual maximal fluctuations range from 11 to 53% for beta power and 12–57% for gamma power.

Among nonlinear measures, DFA and LZC show the largest individual fluctuations, with average maximal deviations of 23% and 10%, respectively (ranging from 5 to 54% for DFA and 4–22% for LZC, depending on the subject). HFD and pMP exhibit the lowest variability, with average maximal deviations of 4% and 6%, respectively. Individual maximal variation ranges from 2 to 8% for HFD and 0–19% for pMP.

When examining individual subjects separately, it is evident that subject S4 shows significantly greater variability, with an average maximal fluctuation of 53% across all measures. In contrast, subjects S1 and S5 exhibit considerably lower variability, with an average maximal fluctuation of 17%. This further emphasizes the strong individuality in EEG measures.

As shown in Fig. 3; Tables 1 and 2, intra-individual annual variation is generally smaller than inter-individual variation, apart from a few exceptions. Notably, in the theta frequency band, subjects S2 and S4 exhibit annual variability comparable in magnitude to that observed between individuals. In subject S4, the variability within the alpha band markedly exceeds inter-individual differences, while in the beta band, it is again of comparable magnitude. For most nonlinear measures (HFD, LZC, DFA), intra-individual variation remains lower than the variation across subjects, with no exceptions. However, in the case of pMP, subject S4 again exhibits greater variability than the group.

## Discussion

In this study, we tested whether EEG measures, while differing between individuals, remain temporally stable within the same person across one year, and if nonlinear measures are temporally more stable at the individual level compared to absolute band powers. For this, we investigated the reliability and long-term temporal stability of EEG band powers and nonlinear EEG measures across 12 months in healthy individuals. Our findings largely support the hypothesis of individual temporal stability, though some nuances remain.

A key finding of this study is the strong individual specificity of EEG measures, with each subject’s values remaining tightly grouped within their own subject-specific range. This largely supports the concept that EEG measures may serve as neural fingerprints — remaining principally stable within individuals while differing significantly between them^34–36^ — although some individuals exhibited fluctuations that challenge the assumption of consistent intra-individual stability.

Regarding the reliability of EEG measures, our findings align with previous research^15,33^, showing that lower-frequency bands (theta and alpha) are the most reliable across sessions. Beta power shows only slightly reduced ICCs in a few temporal channels. Gamma power, in turn, shows a more pronounced decrease in reliability in several channels. Nevertheless, reliability remained high overall, with our lowest observed mean ICC being 0.935 (in the gamma band), which is substantially higher than the ICC of 0.77 for absolute power reported by Gudmundsson et al.^33^. Põld et al.^15^ similarly reported ICCs of 0.80 for gamma and 0.87 for alpha relative power, which is consistent with our results. The reduced reliability in the peripheral channels in gamma and beta bands may be explained by low-level tonic EMG activity that spectrally overlaps with these frequency ranges and is not fully removed by standard preprocessing^51–53^. Although we aimed to obtain EEG recordings free of visible artefacts, the potential influence of subtle tonic EMG activity, particularly in high-frequency bands, was not directly investigated in this study. Nevertheless, it should be kept in mind when interpreting gamma and beta activity in longitudinal analyses, especially in muscle-prone regions.

For nonlinear measures, our results also indicate higher reliability than previously reported. Gudmundsson et al.^33^ found an ICC of 0.70 for LZC, and Põld et al.^15^ reported ICCs of 0.81 for HFD and 0.84 for DFA. In contrast, we observed consistently excellent ICCs above 0.96 (95% CI LBs ≥ 0.908) across all EEG channels for all nonlinear measures. Notably, these measures showed minimal differences between channels, suggesting reduced sensitivity to possible slight EMG input and highlighting their robustness across the spatial domain. Although the study by Põld et al.^15^ assessed long-term stability over three years, the use of only two recordings per subject may have contributed to slightly lower ICCs. Gudmundsson et al.^33^ included 19 recordings over two months, but the older age of participants could have increased intra-individual variability. Our study, using monthly recordings over one year in a younger cohort, showed that nonlinear measures remained highly reliable across all sessions, reinforcing their potential for individualized longitudinal monitoring.

While all EEG measures demonstrated excellent test–retest reliability in all or most channels, high reliability does not necessarily equate to high temporal stability. Therefore, we separately quantified intra-individual variation by calculating the maximum relative differences from each subject’s mean across 12 monthly recordings. This allowed us to directly assess how much a person’s EEG measure fluctuated over time, regardless of between-subject differences. These analyses revealed that although many participants demonstrated stable EEG patterns, a few (most notably participant S4) exhibited fluctuations over time that were comparable to or greater than the variability observed between individuals. Thus, EEG measures cannot universally be assumed to be temporally stable at the individual level, even if group-level reliability appears excellent.

While methodological aspects, such as recording conditions and electrode placement, were carefully controlled, intrinsic physiological factors still contribute to variability. Individual differences in hormonal levels, neuroanatomy, and overall brain physiology may result in varying degrees of natural fluctuation in EEG measures. Additionally, lifestyle factors such as sleep patterns, diet, and physical activity can subtly modulate EEG signals, affecting their stability over time^40^.

Although subject S4 was considered healthy by self-report at the time of the study, such variability may still reflect transient changes in mental state or the early signs of psychological shifts that were not yet subjectively perceived. Psychological states and mental health conditions are known to affect EEG patterns, as shown in previous group studies^4–13^. High levels of stress, anxiety, depression, and other mental states or psychiatric disorders are known to alter brain activity patterns, potentially leading to deviations from typical EEG signatures. Identifying the sources of EEG variability — whether due to intrinsic traits, temporary states, or early pathological changes — will be critical for tailoring analysis strategies.

Equally important is the ability to estimate, in advance, the expected range of normal variability for a given individual. Achieving this requires identifying the key individual factors that contribute to greater variability in EEG measures in the healthy state. Such person-specific variability profiles could help distinguish between brain disorder-related fluctuations and those indicative of normal neuropsychological changes. In future applications, developing heuristics to detect high-variability profiles without the need for long-term tracking will enhance efficiency and individualization. In high variability cases, alternative EEG measures or a combination of measures for individualized baseline approaches may be required.

The second hypothesis proposed that nonlinear EEG measures would exhibit better intra-individual temporal stability than traditional band power measures. Our results strongly support this hypothesis.

While all EEG measures demonstrated excellent test–retest reliability in channel P3, intra-individual temporal stability in the same channel, assessed as maximum relative difference from the individual’s mean, was substantially smaller for nonlinear measures. For instance, mean deviations across subjects for theta and alpha power were 66% and 64%, respectively, compared to only 4% for HFD and 6% pMP.

These results are further supported by findings from Põld et al.^15^, who observed very low relative changes in nonlinear measures at a group level in a test-retest study over three years: 0.18% for HFD and 0.49% for DFA. In comparison, their relative band power measures showed relative changes from 0.72% up to 2.28%. The fact that nonlinear measures in our study showed such small variability even across 12 sessions strengthens the conclusion that they are temporally more stable than traditional band power measures. Põld et al.^15^ demonstrated that nonlinear measures are not only reliable but also temporally more stable at the group level. The present study confirms that these measures are likewise both reliable and highly temporally stable at the individual level. In contrast, band power measures appear more vulnerable to transient fluctuations and may not provide reliable baselines for individual monitoring.

Current findings highlight the importance of an individualized approach to EEG interpretation, moving beyond reliance on fixed population-level norms. Rather than comparing individuals to group averages, establishing person-specific baselines under stable conditions allows for more accurate identification of meaningful neural changes versus natural fluctuations^56^. Our results emphasize that such individualized baselining is essential for reliable longitudinal monitoring. Notably, nonlinear EEG measures provide a particularly strong foundation for this approach, as they exhibit greater resistance to temporal variability than traditional band power measures. This stability makes them promising candidates for biomarkers intended to track brain function over extended periods.

Despite the strong temporal stability observed, the small sample size (nine male participants) limits the generalizability of our findings. Future studies should validate these results in larger, more diverse populations and assess how EEG stability is affected by factors such as age, sex, and individual differences in cognitive functioning. Additionally, a clinically applicable EEG measure must balance long-term stability with sensitivity to dynamic physiological states. Future research should explore this balance to determine which EEG measures are most suitable for clinical applications. Since various biological and lifestyle-related factors can influence natural variability in EEG measures, it is essential to account for individual-specific differences, even in the absence of overt psychological stress or neurological conditions. Deviations from a healthy psychological state and overall mental well-being are precisely the types of changes that are intended to be detected through the establishment of a baseline for EEG variability. Even when working with self-reported healthy subjects, future protocols should include a clinician-led screening to confirm the absence of neurological or psychiatric conditions. Future work should also establish how segment length influences stability and sensitivity of single-channel EEG measures. Varying window sizes will clarify the minimum duration that still yields stable resting-state estimates, and whether longer windows narrow or widen the normative range. Finally, as all recordings were conducted in controlled laboratory conditions, it remains unclear how real-world factors (e.g., time of day, environmental stressors, or diet) influence EEG stability. Future studies should assess EEG reliability in naturalistic settings to improve its applicability for longitudinal monitoring.

## Conclusion

This study confirmed that EEG band power measures are highly reliable over long-term recordings and that nonlinear measures demonstrate comparable levels of reliability. However, nonlinear measures showed greater temporal stability across sessions, making them potentially more suitable for assessing brain state over time, provided they also demonstrate sufficient sensitivity to meaningful neural changes. These findings support the use of nonlinear EEG measures in individualized, longitudinal monitoring frameworks. Furthermore, establishing personalized baselines, rather than relying on normative population averages, appears essential for accurate interpretation of EEG data. Given the overall high reliability across EEG channels, researchers have flexibility in channel selection, although peripheral channels may be best avoided to minimize the influence of artifacts.

## Supplementary Information

Below is the link to the electronic supplementary material.

Supplementary Material 1

## Data Availability

The raw EEG data generated and analyzed during the current study are not publicly available due to data protection and ethical restrictions. However, derived data supporting the findings of this study (including computed measures) are available from the corresponding author upon reasonable request.
