# Supplementary material for: Individual stability of single-channel EEG measures over one year in healthy adults
Source: Sci Rep. 2025 Aug 4;15:28426. doi: 10.1038/s41598-025-13614-y (PMC12322233; doi:10.1038/s41598-025-13614-y)
Supplement: Supplementary file 1 — Supplementary Material 1 [file 41598_2025_13614_MOESM1_ESM.docx]

**Long-term individual stability of single-channel EEG measures in healthy adults**

Tuuli Uudeberg, Laura Päeske, Hiie Hinrikus, Jaanus Lass, Toomas Põld, Maie Bachmann

Supplementary Table S1**.** Intraclass correlation coefficients (ICCs) for EEG measures across 12 monthly recordings per subject (*n* = 9). Values are shown for absolute power in theta, alpha, beta, and gamma frequency bands, as well as for nonlinear measures: Higuchi’s fractal dimension (HFD), Lempel–Ziv complexity (LZC), detrended fluctuation analysis (DFA), and in-phase Matrix Profile (pMP). ICCs for which the lower bound of the 95% confidence interval exceeded 0.9 are shown in bold, reflecting our classification of excellent reliability.

|  | Theta | Alpha | Beta | Gamma | HFD | LZC | DFA | pMP |
| --- | --- | --- | --- | --- | --- | --- | --- | --- |
| O2 | **0.986** | **0.964** | **0.980** | 0.931 | **0.990** | **0.975** | **0.986** | **0.961** |
| O1 | **0.992** | **0.980** | **0.981** | 0.947 | **0.992** | **0.978** | **0.988** | **0.966** |
| Oz | **0.992** | **0.970** | **0.983** | **0.966** | **0.992** | **0.981** | **0.989** | **0.960** |
| Pz | **0.990** | **0.974** | **0.981** | **0.971** | **0.996** | **0.982** | **0.987** | **0.976** |
| P4 | **0.979** | **0.970** | **0.980** | **0.990** | **0.996** | **0.988** | **0.993** | **0.978** |
| CP4 | **0.992** | **0.988** | **0.992** | **0.985** | **0.997** | **0.991** | **0.995** | **0.984** |
| P8 | **0.989** | **0.980** | **0.982** | 0.951 | **0.996** | **0.988** | **0.993** | **0.981** |
| C4 | **0.993** | **0.995** | **0.995** | **0.988** | **0.996** | **0.992** | **0.997** | **0.991** |
| TP8 | **0.986** | **0.988** | 0.908 | 0.863 | **0.980** | **0.978** | **0.994** | **0.984** |
| T8 | **0.991** | **0.991** | 0.951 | 0.786 | **0.985** | **0.976** | **0.992** | **0.990** |
| P7 | **0.991** | **0.981** | **0.986** | 0.941 | **0.993** | **0.985** | **0.994** | **0.987** |
| P3 | **0.981** | **0.975** | **0.988** | **0.987** | **0.996** | **0.988** | **0.994** | **0.978** |
| CP3 | **0.989** | **0.988** | **0.987** | **0.986** | **0.997** | **0.991** | **0.995** | **0.986** |
| CPZ | **0.993** | **0.979** | **0.981** | **0.977** | **0.996** | **0.988** | **0.993** | **0.977** |
| CZ | **0.991** | **0.986** | **0.979** | **0.988** | **0.996** | **0.989** | **0.993** | **0.974** |
| FC4 | **0.992** | **0.988** | **0.990** | **0.986** | **0.996** | **0.992** | **0.995** | **0.983** |
| FT8 | **0.996** | **0.991** | **0.965** | 0.756 | **0.985** | **0.970** | **0.995** | **0.975** |
| TP7 | **0.992** | **0.990** | 0.917 | 0.894 | **0.988** | **0.984** | **0.992** | **0.989** |
| C3 | **0.990** | **0.992** | **0.985** | **0.991** | **0.997** | **0.990** | **0.995** | **0.988** |
| FCz | **0.991** | **0.985** | **0.984** | **0.985** | **0.996** | **0.992** | **0.992** | **0.972** |
| Fz | **0.992** | **0.986** | **0.988** | **0.985** | **0.996** | **0.992** | **0.994** | **0.972** |
| F4 | **0.991** | **0.985** | **0.990** | **0.984** | **0.995** | **0.990** | **0.992** | **0.974** |
| F8 | **0.995** | **0.987** | **0.990** | 0.871 | **0.993** | **0.985** | **0.988** | **0.970** |
| T7 | **0.995** | **0.992** | **0.962** | **0.967** | **0.978** | **0.967** | **0.994** | **0.979** |
| FT7 | **0.996** | **0.989** | **0.985** | 0.842 | **0.990** | **0.981** | **0.993** | **0.974** |
| FC3 | **0.992** | **0.988** | **0.985** | **0.991** | **0.996** | **0.991** | **0.994** | **0.976** |
| F3 | **0.992** | **0.985** | **0.988** | **0.966** | **0.995** | **0.990** | **0.993** | **0.972** |
| FP2 | **0.992** | **0.985** | **0.973** | 0.849 | **0.993** | **0.981** | **0.989** | **0.977** |
| F7 | **0.995** | **0.986** | **0.992** | 0.874 | **0.994** | **0.982** | **0.987** | **0.973** |
| FP1 | **0.993** | **0.985** | **0.985** | 0.867 | **0.993** | **0.981** | **0.988** | **0.975** |
| Mean | 0.991 | 0.984 | 0.978 | 0.935 | 0.993 | 0.985 | 0.992 | 0.977 |
